# Measurement and analysis of standing spine–pelvis–lower limb biomechanical parameters in patients with knee osteoarthritis based on principal component analysis

**DOI:** 10.3389/fbioe.2026.1756378

**Published:** 2026-05-29

**Authors:** Yicong Bai, Yongwang Zhang, Youyue Pang, Guohua Yu, Chang Wang, Mengyue Liu, Shuangqing Du

**Affiliations:** 1 Hebei University of Chinese Medicine, Shijiazhuang, China; 2 Hebei Provincial Hospital of Traditional Chinese Medicine, Shijiazhuang, China

**Keywords:** biomechanics, compensatory mechanism, knee osteoarthritis, lower limb alignment, principal component analysis

## Abstract

**Objective:**

To investigate the Standing Spine–Pelvis–Lower Limb Biomechanical Parameters alignment patterns in patients with varus-type and neutral-type knee osteoarthritis (KOA), to examine the biomechanical characteristics across different sexes, and to provide new directions and theoretical support for the prevention and treatment of KOA.

**Methods:**

A retrospective analysis was conducted on 308 patients with KOA who attended the Department of Orthopaedics III, First Affiliated Hospital of Hebei University of Chinese Medicine, between 1 October 2023 and 31 December 2024. The cohort included 68 males and 240 females, aged 44–85 years, with a mean age of 63 ± 8.24 years. From the clinical observation records, the following parameters were collected: hip-knee-ankle angle (HKA), mechanical axis deviation (MAD), Joint Line Convergence Angle (JLCA), Lateral Proximal Femoral Angle (LPFA), mechanical medial proximal tibial angle (mMPTA), Lumbar Lordosis (LL), Sacral Slope (SS), and baseline demographic data.

**Results:**

No significant differences were observed between varus-type and neutral-type patients in LPFA or SS (P > 0.05). However, principal component analysis revealed differences in the overall biomechanical models. Although no sex-related differences were found in the overall biomechanical model, the parameter compositions driving secondary principal components exhibited gender-specific characteristics. HKA was the leading principal component across all groups, and LPFA consistently served as a major influencing factor. Among varus-type patients, SS was the secondary component in males, whereas LL predominated in females. Among neutral-type patients, LL was the secondary component in males, while mMPTA was the dominant secondary component in females.

**Conclusion:**

Patients with varus and neutral KOA exhibit distinct overall Standing Spine–Pelvis–Lower Limb Biomechanical Parameters alignment patterns and sex-specific biomechanical characteristics. The principal component analysis results reveal different multivariate alignment patterns across subgroups, suggesting possible interactions within the spine–pelvis–lower limb chain, with both sex-specific and type-specific patterns. Therefore, clinical evaluation and treatment of KOA should extend beyond the knee joint itself. LPFA is also critically important, and individualized assessment should be conducted from a holistic biomechanical perspective.

## Introduction

Knee osteoarthritis is a common chronic degenerative joint disease characterized by articular cartilage degeneration and osteophyte formation, and is one of the leading causes of disability among middle-aged and elderly individuals. Globally, approximately 595 million people are affected by OA (about 7.6% of the global population), with the knee joint being the most commonly involved site. Among individuals aged over 40 years, the global prevalence is approximately 22.9% (Mohamed and Alatawi, 2023; Alkalash et al., 2023). The onset of KOA is associated with factors such as age, obesity, genetics, and joint injuries. In recent years, biomechanical factors have attracted increasing attention. Abnormal lower limb alignment (e.g., varus deformity) leads to altered load distribution across the knee joint, accelerating joint degeneration. Clinical studies have demonstrated that alignment correction procedures (such as high tibial osteotomy) can effectively alleviate symptoms in early-stage KOA, further confirming the importance of the biomechanical environment in KOA management (Reichenbach et al., 2022).

However, traditional studies have predominantly focused on single biomechanical parameters, overlooking the integrated coordination between the lower limb and the spine–pelvis complex (Saxby et al., 2016; Prodromos et al., 1985). Principal component analysis (PCA), a multivariate statistical method, can extract major patterns of variation from high-dimensional datasets and reveal intrinsic associations among parameters, making it particularly suitable for analyzing complex biomechanical systems. PCA is a multivariate dimensionality-reduction technique specifically designed to address these limitations. PCA transforms a set of correlated variables into a smaller number of uncorrelated principal components that capture the major sources of variation in the data. This approach eliminates multicollinearity, reduces data redundancy, and reveals intrinsic biomechanical integration patterns that are not observable through separate analyses of individual parameters. Despite its advantages, PCA has rarely been applied to study the spine–pelvis–lower limb alignment in KOA patients. Based on imaging data from 308 KOA patients, this study utilized PCA to systematically analyze lower limb alignment characteristics according to KOA subtype and sex, with the aim of providing a theoretical basis for individualized KOA treatment.

Current treatment follows a stepwise strategy that includes basic lifestyle interventions, rehabilitation training, pharmacological therapy (such as nonsteroidal anti-inflammatory drugs), and surgical treatment for end-stage disease (such as joint replacement). However, pharmacological therapy can only alleviate symptoms and cannot reverse disease progression, and it is associated with adverse effects. Although surgical treatment is effective for end-stage patients, it is highly invasive and costly. Therefore, elucidating the underlying mechanisms of KOA onset and progression, and subsequently developing early intervention and precision treatment strategies, has become an urgent research need.

Although the importance of overall lower limb alignment has been widely recognized, current research and clinical practice still present notable limitations: a lack of holistic analysis, unclear research priorities, and the absence of differentiated focus across patient subtypes.

## Methods

### Source of cases

This study collected data from 331 patients with KOA who underwent full-length weight-bearing radiographs of both lower limbs and the lumbar spine at the Department of Orthopaedics, First Affiliated Hospital of Hebei University of Chinese Medicine (Hebei Provincial Hospital of Traditional Chinese Medicine) between 1 October 2023 and 31 December 2024. Because the proportion of valgus-type KOA patients was low and the sample size insufficient, only neutral-type and varus-type patients with an HKA angle <183° were included (MacDessi et al., 2021). Based on the inclusion and exclusion criteria, imaging and clinical data from 308 patients were ultimately analyzed. Patients with HKA ≤177° were assigned to the varus group, and those with 183° > HKA >177° were assigned to the neutral group. The study was approved by the Institutional Ethics Committee (Approval No.: HBZY2022-KY-068-0168-01). All participants provided written informed consent, and the study adhered to the principles of the Declaration of Helsinki.

### Inclusion criteria

1. Meeting the diagnostic criteria for KOA (Nelligan, 2023): ① recurrent knee pain for more than 1 month; ② age ≥40 years; ③ (standing or weight-bearing) radiographs showing joint space narrowing, subchondral sclerosis and/or cystic changes, and marginal osteophyte formation; ④ morning stiffness ≤30 min; ⑤ crepitus during movement. Diagnosis of KOA was established when criterion ① was met along with any two of criteria ②, ③, ④, or ⑤.
2. Meeting the radiographic diagnostic criteria for the hip-knee-ankle angle (MacDessi et al., 2021), confirmed by full-length weight-bearing radiographs, with HKA ≤183°.
3. Complete data in the case report form.
4. Full-length weight-bearing radiographs of both lower limbs performed at our institution.

### Exclusion criteria

1. History of surgery involving the lumbar spine, hip, knee, or ankle (such as total knee arthroplasty, total hip arthroplasty, or open lumbar spinal surgery).
2. Patients whose radiographic positioning did not meet the predefined standards, who had poor imaging quality, or whose images failed to meet quality requirements during subsequent evaluation.

### Observational indicators

**FIGURE 1:**
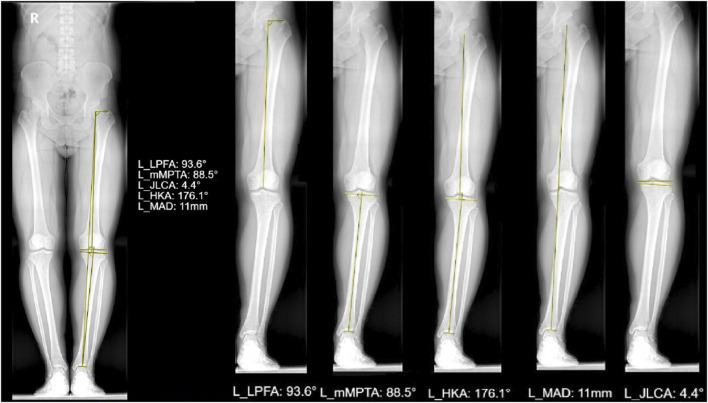
Measurement indices and measurement results (LPFA, mMPTA, HKA, MAD, JLCA).

**FIGURE 2:**
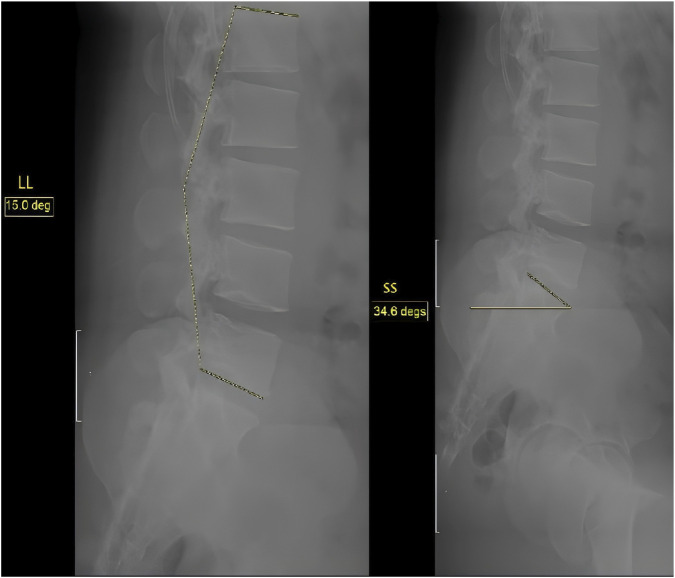
Measurement indices and measurement results (LL and SS).

General Information: Patient age, sex, height, weight, and other baseline data were obtained from the hospital electronic medical record system. The measured angles (JLCA, HKA, LPFA, mMPTA, LL, SS) are illustrated in Figures 1, 2.

Imaging parameters:

Frontal:

Joint Line Convergence Angle (JLCA): the angle between the proximal tibial joint line and the distal femoral joint line.
Hip-Knee-Ankle Angle (HKA): the angle between the line connecting the femoral head center and the midpoint of the femoral condyles, and the line connecting the midpoint of the femoral intercondylar notch and the center of the talus.
Lateral Proximal Femoral Angle (LPFA): the lateral angle formed by the line connecting the femoral head center and the highest point of the greater trochanter, and the line connecting the femoral head center to the knee joint center (femoral mechanical axis).
The medial proximal tibial angle (mMPTA) was defined as the medial angle between the mechanical axis of the tibia and the line connecting the medial and lateral margins of the tibial plateau.

Lateral:

The lumbar lordosis angle (LL) was measured using the Cobb method, namely, the angle between the perpendiculars to the extended lines of the upper endplate of L1 and the lower endplate of L5.
The sacral slope (SS) was defined as the angle between the extended line of the upper endplate of S1 and the horizontal reference line.

### Photography method

**FIGURE 3:**
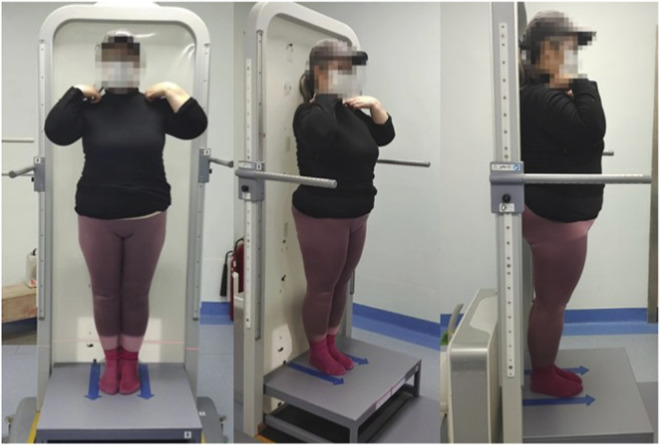
Standardized imaging posture.

The lumbar spine combined with full-length bilateral lower extremity weight-bearing anteroposterior radiographic method is shown in Figure 3. An Optima XR646 HD digital medical X-ray system was used (Biomedical Safety and Standards, 2025). The examination table was adjusted to the standing vertical position, and the standing support plate was positioned at the highest adjustable level (approximately 35 cm above the ground). The distance between the focal spot of the X-ray tube and the detector was set at 100 cm. The exposure range extended from the upper margin including the L1 vertebral body to the lower margin including the ankle joints. The patient stood with the back against the examination table and faced the X-ray tube in an upright posture, with both hands placed on shoulders, feet together, patellae and toes pointing straight forward, lower limbs fully extended, and the posterior lower-limb margins closely contacting the examination table. The patient remains in the same position during a 90°rotation. The affected lower limb, which experiences more severe pain, is brought closer to the radiography table for positioning. A panoramic radiographic mode was used, in which the X-ray tube automatically exposed from top to bottom while the detector moved synchronously from top to bottom. The system automatically stitched the continuous exposures to generate a full-length bilateral lower extremity weight-bearing radiograph combined with a lumbar spine radiograph. Compared with imaging obtained in a natural posture, this standardized fixed posture reduced measurement error for subsequent analyses.

### Measurement methods

HKA, MAD, JCLA, LPFA, and mMPTA were all measured using automatic measurements provided by United Imaging Intelligent AI software (Meng et al., 2022). Four individuals recorded the output from the software, and four individuals verified the data. LL and SS were measured using the built-in ruler and angle measurement tools in the MI Platform software (CNKI, 2015). Measurements were conducted by three members of the research team based on fixed anatomical landmarks. All intragroup correlation coefficients exceeded 0.85 (ICC >0.85). For all parameters, the mean value of the three measurements was used for statistical analysis.

### Statistical analysis

SPSS 26.0 and GraphPad Prism 10 software were used for data processing and statistical analyses. Continuous variables were tested for normality. Normally distributed variables are presented as mean ± standard deviation (x ± s) and compared using the independent-samples t-test. Non-normally distributed variables are presented as median (interquartile range) [M (P25, P75)] and compared using the Mann–Whitney U test (a nonparametric test for two-group comparisons). Categorical variables were analyzed using the chi-square test (Chi-Square Test). A significance level of α = 0.05 was adopted, and P < 0.05 was considered statistically significant. PCA was applied for data analysis, components with eigenvalues greater than 0.8 were retained based on the correlation matrix.

PCA is a multivariate dimensionality-reduction technique that transforms a set of potentially correlated variables into a smaller number of uncorrelated principal components (PCs) . In this study, PCA was applied to the six alignment parameters (HKA, LPFA, mMPTA, JLCA, LL, and SS) to identify latent patterns of correlated anatomical variation that characterize the integrated spine–pelvis–lower limb biomechanical configuration.

## Results

General characteristics A total of 308 patients with KOA were eventually included, comprising 198 patients with varus deformity (64.3%) and 110 patients with neutral alignment (35.7%). For patients with bilateral involvement, only the more symptomatic limb was included. Detailed information is presented in Table 1.

**TABLE 1:** General characteristics.

Observation indicators	Varus group	Neutral group	F value or χ^2^ value	P value
Number of cases (n)	198	110	-	-
Age (M (P25, P75), years)	65 (59,70)	62 (55.75,69.25)	−1.922	0.055
Sex (n, male:female)	44:154	24:86	0.007	0.935
Height (M (P25, P75), cm)	160 (158,165)	161 (158,165)	−1.163	0.244,763
Weight (M (P25, P75), kg)	67 (60.75,75)	67.5 (60.75,75)	−0.194	0.846,077
Bmi (x ± s, kg/cm2)	26.1 ± 3.1	25.7 ± 3.4	0.676	0.411,758
Disease duration (M (P25, P75), years)	2 (1,5)	1.5 (0.6,4.125)	−1.455	0.146

The results of the parameter comparisons showed no significant differences in LPFA, mMPTA, or SS between the two groups (P > 0.05). JLCA, HKA, MAD, and LL demonstrated statistically significant differences (P < 0.01). Detailed results are presented in Table 2.

**TABLE 2:** Calculated results of lower-limb alignment angles, lumbar angles, sacral angles.

Parameter	Varus group	Neutral group	F value or χ^2^ value	P value
LPFA	92.74 ± 5.5	93.95 ± 4.7	3.788	0.053
mMPTA	84.75 (83,86.1)	87.25 (86,88.525)	−9.066	<0.01
JLCA	2.9 (1.8,4.125)	2 (1,2.9)	−4.633	<0.01
HKA	174 (171.3,175.625)	179.3 (178.2,180.65)	−14.543	<0.01
MAD	18 (12,26)	1 (-4.25,4)	−14.402	<0.01
LL	25.4 ± 15.1	31.6 ± 9.0	32.207	<0.01
SS	35.05 (28.35,42.125)	33.1 (29.35,38.4)	−0.893	0.372

LPFA, mMPTA, JLCA, HKA, MAD, lower-limb angles; LL, lumbar lordosis angle; SS, sacral slope.

**FIGURE 4:**
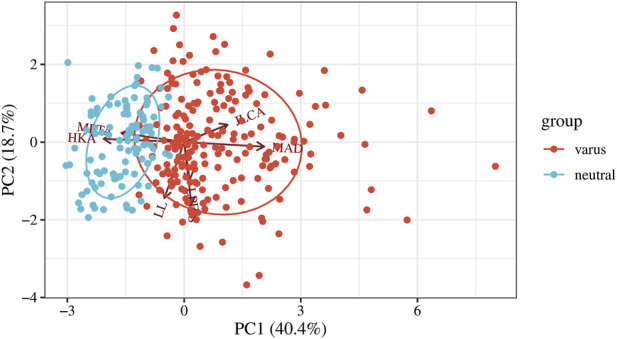
Principal component analysis score plot for the varus and neutral groups. Red circles: male patients (n = 68); blue squares: female patients (n = 240). The segregation along the first principal component (PC1) between the two groups suggests distinct multivariate force line characteristics in patients with inverted-type KOA compared to those with neutral-type KOA.

The score plot of principal component analysis for lower-limb alignment in different subtypes is shown in Figure 4, demonstrating a clear separation between the two groups.

**FIGURE 5:**
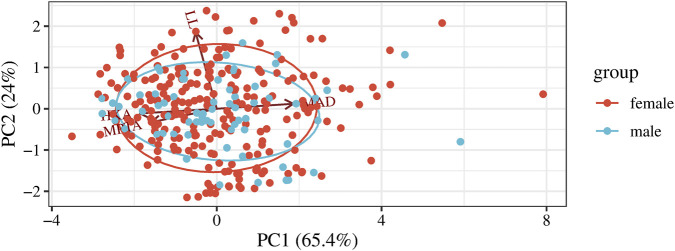
Principal component analysis score plot for different sexes. Red circles: male patients (n = 68); blue squares: female patients (n = 240). PC1 explains 34.2% and PC2 explains 18.6% of the variance. The scatter points of males and females overlap substantially, with no clear separation along either PC1 or PC2, indicating that the overall multivariate biomechanical profile does not differ globally between sexes.

The principal component analysis results for lower-limb alignment stratified by sex are shown in Figure 5. The scatter points overlapped between groups, indicating no apparent differences.

**FIGURE 6:**
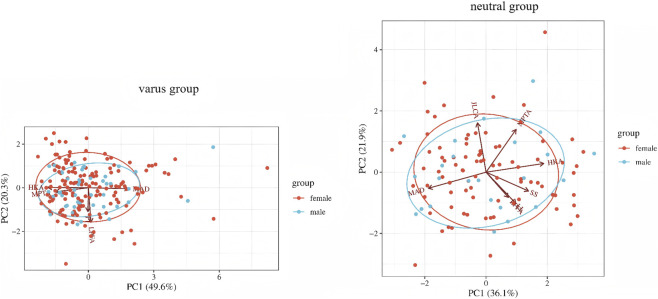
Principal component analysis score plot for both sexes. Varus group: males (red circles, n = 44) vs. females (blue squares, n = 154). Neutral group: males (red circles, n = 24) vs. females (blue squares, n = 86). In both subtypes, the scatter points overlap considerably, indicating that within the same alignment type, the overall multivariate alignment profile does not differ substantially between sexes.

The principal component analysis of lower-limb alignment in male and female patients in the varus group and that in the neutral group is shown in Figure 6. The scatter points overlapped in both groups, indicating no apparent differences.

**FIGURE 7:**
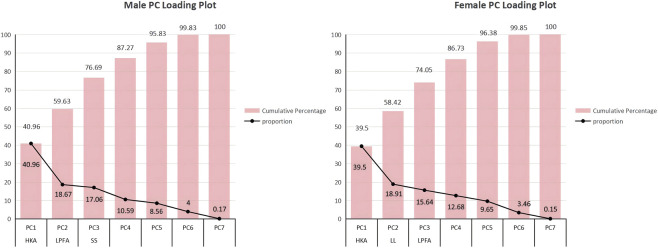
Principal component loading plot for the varus group. The plot shows the contribution of each alignment parameter to the first two principal components (PC1 and PC2). In both males and females of the varus group, HKA is the dominant variable on PC1. For males, LPFA is the secondary component, loading heavily on PC2; for females, LL is the secondary component, loading heavily on PC2. The variance explained by PC1 and PC2 is indicated on the axes.

For patients in the varus group, the principal component characteristic parameters were HKA, LPFA, and SS in males, and HKA, LL, and LPFA in females, as shown in Figure 7.

**FIGURE 8:**
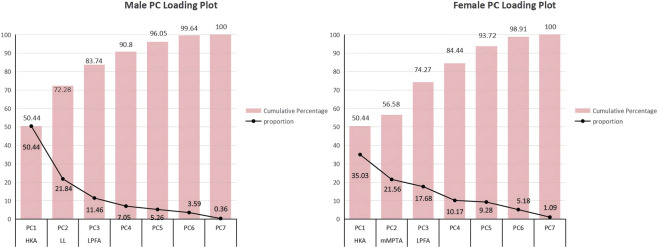
Principal component loading plot for the neutral group. The plot shows the contribution of each alignment parameter to the first two principal components (PC1 and PC2). In both males and females of the neutral group, HKA remains the dominant variable on PC1. For males, LL is the secondary component, loading heavily on PC2; for females, mMPTA emerges as the dominant secondary component, with notable loading on PC2. The variance explained by PC1 and PC2 is indicated on the axes.

For patients in the neutral group, the principal component characteristic parameters were HKA, LL, and LPFA in males, and HKA, mMPTA, and LPFA in females, as shown in Figure 8.

## Discussion

KOA is a degenerative disease in which degeneration occurs not only in the knee joint but also in joints such as the lumbar spine and pelvis. The multiple mechanical joints of the human body form an integrated kinetic chain. In patients with knee osteoarthritis, mechanical transmission from the pelvis and lumbar spine to the knee should be considered in addition to the local mechanical load on the knee joint. The spine, pelvis, and lower limbs constitute a closely linked biomechanical system that plays a crucial role in maintaining upright posture, walking, and load transmission. Previous studies have indicated that a close biomechanical relationship exists among the spine, pelvis, and lower limbs. Lumbar spine changes in patients with KOA are important factors influencing knee joint pathology. Mechanical alignment transmits and distributes forces from the spine to the pelvis and subsequently to the lower limbs, involving interactions across multiple joints (Zhang et al., 2025; Du et al., 2024).

As a bony structure connecting the spine and the lower limbs, the pelvis transfers the load from the upper body to both acetabula, and subsequently through the femur, tibia, and other lower-limb bones to the ground (Raad, 2015). Changes in sagittal spinal curvature (such as increased lumbar lordosis) directly influence pelvic tilt through alterations in sacral slope. Pelvic tilt results in asymmetric loading of both hip joints, which subsequently affects load distribution and movement patterns of the femur, knee joint, and ankle joint, forming a chain biomechanical effect from the pelvis to the lower limbs. Several studies have further indicated that limited ankle dorsiflexion can, in turn, influence anterior pelvic tilt, thereby forming a closed-loop mechanical feedback system (Vialle et al., 2005; van Erp et al., 2022; Bley et al.; Rao et al., 2024).

This study innovatively applied principal component analysis to extract major variation patterns from extensive imaging data, reduce computational burden, eliminate noise, and visualize complex skeletal changes (Cross et al., 2014). By simultaneously considering multiple mechanical angle parameters and treating the mechanical influences on the human body as an integrated whole, the mechanical parameters of KOA patients with different alignment types were compared comprehensively and visualized for direct comparison. The PCA results indicated that the core biomechanical composition differed substantially across KOA subtypes (Their primary components differ in both composition angle and sequential orderas shown in Figures 7, 8). Suggesting that mechanical abnormalities in KOA represent systemic rather than isolated alterations. Therefore, systematic evaluation from a multidimensional perspective rather than reliance on a single parameter is warranted.

This study measured the mechanical parameters of the lumbar spine, pelvis, and lower limbs and compared these parameters between the varus and neutral KOA groups. Because the primary grouping criterion was knee joint deformity, local knee parameters such as HKA and mMPTA typically differed between the two groups, whereas non-knee parameters such as SS and LPFA did not. A comparison based solely on individual parameters was insufficient to determine differences in overall biomechanics. The principal component analysis score plots demonstrated that, under the combined influence of multiple mechanical parameters, the overall loading conditions differed distinctly between the two groups, indicating that the integrated biomechanical profiles of varus and neutral KOA patients were not identical. Meanwhile, we can view the measured parameters and the results obtained from PCA (such as score plots, principal component characteristics) as a “biomechanical model”, thereby revealing the potential patterns of related anatomical variations and reflecting the overall biomechanical load. It is important to emphasize that this statistical construct does not represent a deterministic mechanical simulation, but rather a data-driven representation of how the spine, pelvis, and lower limb segments functionally co-vary in the presence of knee pathology.

Although the overall biomechanical loading differed between the two KOA alignment subtypes, Figures 5, 6 showed that such biomechanical differences were not evident across sexes. Despite substantial anatomical differences in the pelvis between males and females, no sex-related differences were observed when multiple mechanical parameters were integrated into a unified analysis. However, decomposition of the loading matrices (Figures 7, 8) revealed distinct internal architectures of the biomechanical model stratified by sex. Specifically, while the overall variance structure is similar, the specific combinations of anatomical parameters that drive the secondary axes of variation (PC2 and PC3) differ between males and females. This indicates that the covariance networks adapt in a sex-specific manner, even if the gross alignment output appears similar.

Although the overall biomechanical profile was similar between males and females, the primary contributing components differed. As shown in Table 3; Figures 7, 8, these exploratory findings raise the hypothesis that, in varus KOA, SS may be more relevant to the alignment pattern in males and LL more relevant in females; in neutral KOA, LL may be more relevant in males and mMPTA more relevant in females. These differences may be related to sex-specific physiological differences in pelvic morphology, lumbar curvature, and muscle strength. Mirahmadi et al. (2024) reported that females generally have a wider pelvis and a greater anterior pelvic tilt, leading to increased femoral adduction and consequently a greater knee adduction moment, which elevates medial compartment loading. The cross-sectional area of core trunk muscles (such as the erector spinae, psoas major, and quadratus lumborum) is significantly larger in males than in females, whereas the pelvic structure in females provides improved stability but may also result in greater lumbar stress under comparable loading conditions due to the relative positioning of the pelvis and lumbar spine (Oliva-Lozano and Muyor, 2020; Tanaka et al., 2023). Therefore, for male KOA patients, greater attention should be given to pelvic parameters, whereas female patients should be monitored more closely for lumbar degenerative changes that may influence knee joint biomechanics.

**TABLE 3:** Principal component characteristic angles of lower-limb alignment in KOA patients across sex and alignment subtypes.

Sex	PC1	PC2	PC3
Varus type			
Male	HKA	LPFA	SS
Female	HKA	LL	LPFA
Neutral type			
Male	HKA	LL	LPFA
Female	HKA	mMPTA	LPFA

Patients of different sexes and alignment types show different multivariate alignment patterns that may reflect adaptive strategies, suggesting that clinical evaluation of KOA should adjust therapeutic targets according to sex-specific compensation patterns. In surgical interventions for KOA (such as osteotomy or total knee arthroplasty), individualized treatment strategies should be developed based on each patient’s compensatory characteristics. Although surgical treatment can effectively relieve joint pain in KOA, postoperative satisfaction remains suboptimal. Evidence indicates (Batailler et al., 2021) that patient satisfaction after TKA (TotalKneeArthroplasty)ranges only from 75% to 89%, and some patients may require revision surgery within 5 years after primary TKA. To further improve surgical outcomes, reconstruction of the global spine–pelvis–lower limb biomechanical balance should be considered, shifting from a “local treatment approach”to a “holistic perspective.”Systematic evaluation of abnormalities along the entire biomechanical chain would enable more accurate diagnosis and surgical planning. Marti et al. (Marti et al., 2004) suggested that, during HTO planning, attention should be paid to coronal alignment correction while controlling variations in sagittal parameters to prevent unintended sagittal malalignment that may cause abnormal knee biomechanics and thereby improve long-term surgical outcomes.

For patients with knee osteoarthritis (KOA), previous studies have primarily focused on the HKA angle. In contrast, the present study demonstrates through sex-stratified analyses that the LPFA plays an important role across different sexes and KOA subtypes. This finding shows that LPFA is consistently associated with the principal component structure across all patient subgroups, and this association remains present regardless of sex-related differences in pelvic morphology. The observation that HKA is the leading principal component is not surprising, as it reflects the grouping variable. More informative is the finding that LPFA consistently appears as an influential factor across subgroups, independent of HKA. LPFA may constitute an important compensatory adjustment made by the body in response to abnormal knee joint alignment. Moreover, the hip joint serves as an intermediary link through which the lumbar spine and pelvis influence the lower extremities. When changes occur in the lumbar spine (LL) and pelvis (SS)—including but not limited to spinal degeneration, vertebral fusion surgery, or hip arthroplasty—the direction of loading on the hip joint undergoes corresponding adjustments to maintain trunk balance (Li et al., 2024; Rice et al., 2024; Harada et al., 2022).

This study has several limitations. First, complete baseline data for patients with valgus deformity were lacking, resulting in an incomplete characterization of lower-limb alignment features in this subgroup. Future studies should expand the sample size to address this gap. Second, the study focused exclusively on alignment-related indicators and did not incorporate other baseline variables, such as lifestyle habits or physical activity status, into the analysis. Subsequent research may broaden the analytical dimensions to more comprehensively elucidate the pathogenesis and influencing factors of KOA.

## Limitations

This study has several limitations. First, we excluded valgus knee osteoarthritis (KOA) to avoid confounding from opposite mechanical alignment, which limits the generalizability of our findings to the entire KOA population. Second, the hip–knee–ankle angle (HKA) was used both for patient grouping and as an input variable in principal component analysis (PCA). This circularity causes HKA to dominate PC1; therefore, our interpretation focuses on PC2, PC3, and their sex- and subtype-specific patterns. Third, due to the retrospective, cross-sectional design, we did not collect several clinically relevant variables, including radiographic severity (e.g., Kellgren–Lawrence grade), pain severity, functional status (e.g., WOMAC score), laterality (beyond selecting the more symptomatic knee), or physical activity level. These unmeasured factors may act as confounders or effect modifiers. Finally, this was a single-center, retrospective study without external validation. Causal relationships or compensatory mechanisms cannot be inferred from cross-sectional imaging data. Future prospective, multicenter studies with comprehensive clinical data and independent validation are needed.

## Conclusion

Lower-limb alignment characteristics differ across KOA subtypes and between sexes. The principal component analysis reveals that the composition of principal components differs by sex and alignment subtype, suggesting that the spine–pelvis–lower limb alignment patterns are not uniform across patient subgroups. Therefore, clinical evaluation and treatment of KOA should extend beyond the knee joint, as LPFA is equally important. Individualized assessment should be conducted from an integrated biomechanical perspective.

Based on the findings of principal component analysis (PCA), several instances of individualized treatment or prevention can be taken into account. For male patients with a varus - type condition, in which the sacral slope (SS) serves as a secondary principal component, a preoperative evaluation of pelvic alignment is recommended. This is because an abnormal sacral slope may impact the outcome of high tibial osteotomy via pelvic compensation. For female patients with a varus - type condition, where lumbar lordosis (LL) is the dominant factor, an assessment of the lumbar spine is advisable; concurrent lumbar rehabilitation may contribute to optimizing lower - limb loading subsequent to knee surgery. For female patients with a neutral - type condition, in which the medial proximal tibial angle (mMPTA) emerges as a secondary component, even a subtle inclination of the tibial plateau should be monitored to impede the progression towards varus deformity. These examples demonstrate how the proposed PCA - based analytical framework can guide patient - specific clinical decision - making.

## Data Availability

The raw data supporting the conclusions of this article will be made available by the authors, without undue reservation.
